# P2Y12 Inhibitor Pretreatment in Non-ST-Segment Elevation Acute Coronary Syndromes Undergoing a Late Invasive Strategy—A Portuguese Multicenter Nationwide Registry Analysis

**DOI:** 10.3390/biomedicines13092212

**Published:** 2025-09-09

**Authors:** Adriana Vazão, Carolina Miguel Gonçalves, André Martins, Mariana Ferreira Carvalho, Margarida Cabral, Luís Graça Santos, Sidarth Pernencar, João Filipe Carvalho, João Morais, on behalf of the Portuguese Registry on Acute Coronary Syndromes (ProACS) Investigators

**Affiliations:** 1 Unidade Local de Saúde da Região de Leiria, E.P.E., 2410-197 Leiria, Portugal; 2 ciTechCare—Center for Innovative Care and Health Technology, Polytechnique of Leiria, 2411-901 Leiria, Portugal

**Keywords:** P2Y12 inhibitor, NSTE-ACS, pretreatment, antithrombotic therapy, late invasive strategy

## Abstract

**Background/Objectives:** Current guidelines do not specifically address the use of P2Y12 inhibitor (P2Y12i) pretreatment in patients with non-ST-segment elevation acute coronary syndrome (NSTE-ACS) who are expected to undergo a late invasive strategy. Nevertheless, such pretreatment may be considered in patients without a high bleeding risk (Class of Recommendation, IIb; Level of Evidence, C). Despite this ambiguity, P2Y12i pretreatment remains a common clinical practice. The present study aimed to evaluate the in-hospital prognostic impact of P2Y12i treatment prior to coronary angiography (CAG) in NSTE-ACS patients undergoing a late invasive strategy (CAG > 24 h after hospital admission). **Methods:** A retrospective analysis was conducted on NSTE-ACS patients undergoing a late invasive strategy included in the Portuguese Registry on Acute Coronary Syndromes between 2010 and 2023. The primary endpoint was a composite of in-hospital events, including all-cause mortality, non-fatal re-infarction, non-fatal stroke, and heart failure (HF). Secondary endpoints included the individual components of the primary endpoint and major bleeding (BARC types 3 and 4). **Results:** A total of 3776 patients were included (mean age, 66 ± 12 yrs; 29% female), of whom 1530 (41%) received P2Y12i pretreatment (group 1). Group 1 had a lower prevalence of prior myocardial infarction (16% vs. 21%) and prior percutaneous coronary intervention (12% vs. 15%) (both *p* ≤ 0.001). Although obstructive coronary artery disease was more frequent in group 1 (84% vs. 77%, *p* < 0.001), the presence of multivessel disease did not differ (52% vs. 52%, *p* = 0.667). Considering in-hospital antithrombotic therapy, group 1 had higher prescriptions of clopidogrel (68% vs. 56%), aspirin (99% vs. 81%), unfractionated heparin (21% vs. 8%), and enoxaparin (80% vs. 56%) (all *p* < 0.001). There was no significant difference in the primary composite endpoint between groups (9% vs. 9%, *p* = 0.906). Similarly, the secondary endpoints of all-cause mortality (0.6% vs. 0.7%), re-infarction (1.3% vs. 0.7%), stroke (0.7% vs. 0.4%), and HF (7% vs. 8%) did not differ significantly between groups (all *p* > 0.05). Nevertheless, group 1 exhibited higher rates of major bleeding (0.8 vs. 0.2%, OR 3.48, CI 95% 1.22–9.89, *p* = 0.013). **Conclusions:** Pretreatment with a P2Y12i in NSTE-ACS patients undergoing a late invasive strategy was not associated with reduction in the primary endpoint, although it was associated with higher rates of major bleeding.

## 1. Introduction

The prognostic impact of pretreatment with antiplatelet therapy in patients with acute coronary syndromes (ACSs) has been extensively investigated in recent years [1,2,3,4,5,6,7]. The term pretreatment refers to a strategy in which an antiplatelet agent, typically a P2Y12 inhibitor (P2Y12i), is administered orally as a loading dose prior to coronary angiography (CAG), in addition to acetylsalicylic acid. The optimal pretreatment approach varies depending on clinical presentation, particularly between ST-segment elevation myocardial infarction (STEMI) and non-ST-segment elevation acute coronary syndromes (NSTE-ACSs).

In STEMI patients, pretreatment with a P2Y12i has been associated with a reduced risk of stent thrombosis and cardiogenic shock, as well as improved pre-intervention coronary perfusion, without a significant increase in hemorrhagic complications [8]. These findings are derived mainly from observational studies, whereas randomized controlled trials (RCTs) investigating pretreatment with ticagrelor [5] and clopidogrel [2,3] showed outcomes comparable to those observed when treatment was administered at the time of percutaneous coronary intervention (PCI). Reflecting this evidence, current European guidelines downgraded the recommendation for pretreatment in STEMI from Class I to IIb [9].

In the context of NSTE-ACS, several pretreatment strategies have been evaluated. The first randomized data originated from the PCI-CURE trial, which compared pretreatment with clopidogrel to no pretreatment, revealing a benefit regarding major cardiovascular event reduction [1]. It is important to note, however, that the median time from admission to CAG in this trial was 10 days—substantially longer than current practice. In the ACCOAST trial, the use of prasugrel pretreatment compared to administration after CAG in patients proceeding to PCI revealed comparable major ischemic events at the cost of higher incidence of major bleeding in the pretreatment arm [4]. Similar results were obtained in patients enrolled in the DUBIUS trial, which compared a ticagrelor-based pretreatment strategy to no pretreatment. This study revealed non-statistically significant differences in event rates between groups and was terminated prematurely due to a globally low incidence of both ischemic and hemorrhagic events [10].

Taken together, the results of these RCTs suggest no clear clinical benefits of pretreatment in patients expected to undergo an early invasive strategy, i.e., CAG performed in the first 24 h after the diagnosis. Conversely, in the context of a late invasive strategy, pretreatment may be beneficial in selected NSTE-ACS patients, provided that the potential benefit is carefully weighed against the individual bleeding risk [9]. Indeed, current European guidelines consider pretreatment with a P2Y12i in NSTE-ACS patients undergoing a non-early invasive strategy and without a high bleeding risk as a Class IIb recommendation [9].

The present study aimed to describe the in-hospital outcomes among NSTE-ACS patients undergoing a late invasive strategy, comparing those receiving P2Y12i pretreatment with those who did not.

## 2. Materials and Methods

The Portuguese Registry on Acute Coronary Syndromes (ProACS), a project of the Portuguese Society of Cardiology, was initiated in 2002 and is currently coordinated by the National Center for Data Collection in Cardiology. It is a nationwide, multicenter, continuous, prospective observational registry registered at ClinicalTrials.gov (NCT01642329) [11]. The study protocol was approved on 13 September 2010 by the Portuguese Data Protection Authority (Comissão Nacional de Proteção de Dados). All Portuguese cardiology departments were invited to participate in the registry and to consecutively include eligible adult patients hospitalized due to ACS. By 2016, forty-nine Portuguese centers were actively recruiting, covering the entire geographic area of mainland Portugal and the islands.

All consecutive patients included in the ProACS between 1 October 2010 and 31 October 2023 were considered for inclusion.

### 2.1. Patient Selection

Patients were eligible for inclusion if they had a diagnosis of NSTE-ACS and were scheduled to undergo a late invasive strategy, defined as CAG performed > 24 h after hospital admission. Patients already receiving P2Y12i therapy (clopidogrel, prasugrel, or ticagrelor), those with a previous diagnosis of atrial fibrillation (AF) or AF on the admission electrocardiogram (ECG), and those on oral anticoagulation (OAC) (including vitamin K antagonists and direct oral anticoagulants) were excluded. Baseline patient demographic data, cardiovascular risk factors, and clinical, laboratory, echocardiographic, and CAG data were collected. Two cohorts of patients were defined: patients receiving P2Y12i pretreatment (i.e., loading dose followed by maintenance oral dose of either clopidogrel, prasugrel, or ticagrelor) before CAG (group 1) and patients without pretreatment (group 2).

### 2.2. Primary and Secondary Endpoints

The primary endpoint was defined as a composite of in-hospital events, including all-cause mortality, non-fatal re-infarction, non-fatal stroke, and heart failure (HF). In-hospital re-infarction was defined based on de novo clinical signs and symptoms of myocardial infarction (MI) accompanied by electrocardiographic changes and a ≥20% increase in cardiac troponins compared with baseline values [12,13,14]. Stroke was defined as an acute focal injury of the central nervous system resulting in neurological deficit caused by a vascular etiology, including cerebral infarction and intracerebral/subarachnoid hemorrhage [15]. Heart failure was defined as the development of symptoms and/or signs of fluid overload occurring after the diagnosis of ACS and during hospitalization, requiring initiation of decongestive therapy (excluding patients with Killip–Kimball (KK) class > 1 on admission) [16,17,18,19].

Secondary endpoints included the individual components of the primary endpoint (all-cause mortality, non-fatal re-infarction, non-fatal stroke, and HF) plus major bleeding. Major bleeding was defined as per the Bleeding Academic Research Consortium (BARC) definition and included only type 3 and type 4 bleeding [20].

### 2.3. Statistical Analysis

Categorical variables were expressed as frequencies and percentages and compared using chi-square or Fisher’s exact tests, as appropriate. Continuous variables were expressed as mean ± standard deviation or median with interquartile ranges and compared between groups using the independent samples *t*-test or Mann–Whitney U test, depending on distribution.

All analyses were performed using SPSS^®^ software for Windows, version 29.0. All *p*-values were two-sided and were considered statistically significant when <0.05.

## 3. Results

**Figure 1:**
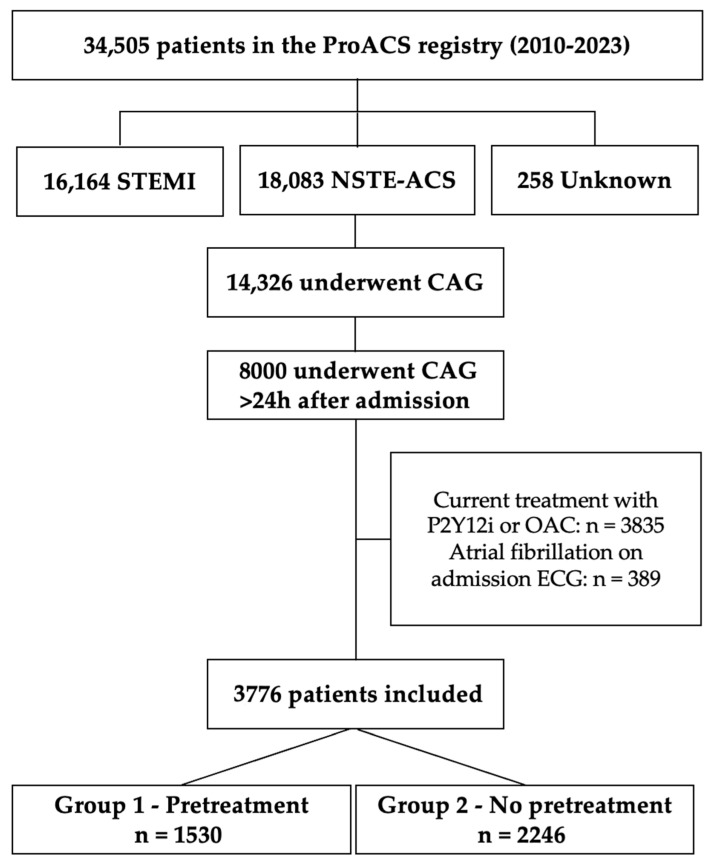
Patient selection (CAG—coronary angiography; ECG—electrocardiogram; NSTE-ACS—non-ST elevation acute coronary syndrome; OAC—oral anticoagulant; P2Y12i—P2Y12 inhibitor; ProACS—Portuguese Registry on Acute Coronary Syndromes; STEMI—ST elevation myocardial infarction).

A total of 34,505 patients were included in the registry during the study period. Of these, 18,083 patients (52.4%) were admitted with NSTE-ACS (including patients diagnosed with non-ST-segment elevation MI [NSTEMI] or unstable angina [UA]); 16,164 (46.8%) with STEMI; and 258 (0.7%) with an undetermined diagnosis. Among NSTE-ACS patients, 14,326 (79.2%) underwent CAG, and 8000 (44.2%) underwent CAG more than 24 h after hospital admission, thus meeting the inclusion criteria for this study. After excluding 3835 patients already receiving P2Y12i or OAC and 389 patients with a history of AF or AF on the admission ECG, a total of 3776 patients were included in the final analysis (Figure 1).

### 3.1. Patient Baseline Characteristics

A total of 3776 NSTE-ACS patients were included in this study: group 1 (pretreatment) comprised 1530 patients (41%), and group 2 (no pretreatment) comprised 2446 patients (59%). Baseline patient characteristics are summarized in Table 1.

The overall mean age was 66 ± 12 years, and 1085 (29%) were female. Group 1 patients had a lower prevalence of dyslipidemia (60% vs. 66%, *p* ≤ 0.001) and chronic coronary heart disease, including prior angina (20% vs. 38%, *p* < 0.001), prior MI (16% vs. 21%, *p* = 0.001), and prior PCI (12% vs. 15%, *p* = 0.001)]. On the other hand, prior history of ischemic stroke or transient ischemic attack was more frequent (7% vs. 5%, *p* = 0.003). Family history of coronary artery disease (CAD) was less frequent in group 1 (5% vs. 7%, *p* = 0.011).

Several differences in admission parameters were observed between groups (Table 2). The most common admission diagnosis was NSTEMI (87%), which was more frequent in group 1 (90% vs. 86%, *p* < 0.001). The predominant presenting symptom was acute chest pain (93%).

On admission, there were no statistically significant differences in KK class, with the majority of patients presenting in class I (90%). Regarding laboratory parameters, group 1 had a slightly higher mean hemoglobin level on admission blood analysis (14 ± 1.8 g/dL vs. 13.8 ± 1.8 g/dL, *p* = 0.003), although both values remained within the normal range. There were no differences in creatinine levels (1.1 ± 0.9 mg/dL vs. 1.1 ± 1, *p* = 0.800) and in platelet counts (220 ± 65 × 10^3^/mm^3^ vs. 220 ± 66 × 10^3^/mm^3^, *p* = 0.497). Regarding echocardiography parameters, patients in group 1 less frequently had left ventricular ejection fraction <50% (20% vs. 24%, *p* = 0.032).

Regarding CAG data (Table 3), the median time from admission to CAG was 2 days, with no difference between groups (*p* = 0.852). Overall, radial artery access was used in 79% of cases and more frequently in group 1 (86% vs. 73%, *p* < 0.001).

Group 1 had higher incidence of obstructive CAD (84% vs. 77%, *p* < 0.001) and more often required repeated CAG during hospitalization (8% vs. 4%, *p* < 0.001). However, the rate of multivessel disease did not significantly differ (52% vs. 52%, *p* = 0.667). Overall, the most common culprit vessel was the right coronary artery (41%), with no difference between groups (45% vs. 38%, *p* = 0.851). Revascularization procedures were also more frequent in group 1, including PCI (63% vs. 60%, *p* = 0.019) and referral for coronary artery bypass graft (13% vs. 10%, *p* = 0.002).

Regarding in-hospital antithrombotic therapy (Table 4), group 1 had higher prescription rates of aspirin (99% vs. 81%), clopidogrel (68% vs. 56%), ticagrelor (38% vs. 28%), unfractionated heparin (21% vs. 8%), and enoxaparin (80% vs. 56%) (all *p* < 0.001). In contrast, fondaparinux (12% vs. 41%, *p* < 0.001) and glycoprotein IIb/IIIa inhibitors (2% vs. 9%, *p* < 0.001) were less frequently prescribed in group 1.

### 3.2. Primary and Secondary Endpoints

**Figure 2:**
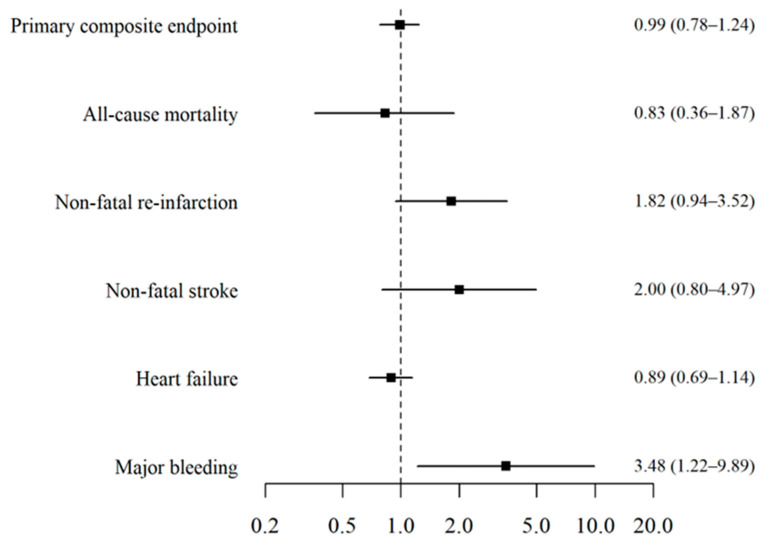
Forest plot for primary and secondary endpoints.

There were no statistically significant differences in the primary composite endpoint (9% vs. 9%, *p* = 0.906). Similarly, the secondary endpoints of all-cause mortality (0.6% vs. 0.7%), re-infarction (1.3% vs. 0.7%), stroke (0.7% vs. 0.4%), and heart failure (7% vs. 8%) were comparable between groups (all *p* > 0.05). Nevertheless, group 1 exhibited higher rates of major bleeding (0.8 vs. 0.2%, OR 3.48, CI 95% 1.22–9.89, *p* = 0.013) (Table 5). These results are further illustrated in the forest plot of primary and secondary endpoints (Figure 2).

## 4. Discussion

In this nationwide Portuguese registry, patients undergoing a late invasive strategy represented 44% of the NSTE-ACS population, a lower proportion than reported in similar studies, which found rates of approximately 60% [21,22]. Among these patients, 41% received pretreatment, also lower than in comparable cohorts, where pretreatment rates were around 74% [21].

The optimal timing for oral P2Y12i administration in NSTE-ACS remains a matter of debate. While earlier initiation may theoretically reduce periprocedural thrombotic complications, it must be weighed against the increased bleeding risk and the potential risk of administering potent antiplatelet therapy to patients ultimately found not to have obstructive CAD—or, in some cases, to those with alternative diagnoses where it may be harmful (e.g., acute aortic syndrome).

The present retrospective registry-based study found that pretreatment with P2Y12i in patients expected to undergo a late invasive strategy was not associated with improved in-hospital outcomes, including all-cause mortality, re-infarction, stroke, or heart failure. In this cohort, a late invasive strategy represented a median time from admission to CAG of just two days—a relatively short interval compared to the PCI-CURE [1] (10 days) yet longer than the median of four hours observed in the ACCOAST trial [4]. In fact, it appears that the shorter the time from admission to CAG, the fewer benefits are observed with pretreatment. Nevertheless, the timeframe in our analysis reflects contemporary clinical practice in real-world settings, particularly within Portuguese hospitals.

Some differences in baseline clinical characteristics were observed in this study. In particular, the lower prevalence of prior MI and prior PCI in the pretreatment group is a curious finding, as those patients are generally at higher risk for recurrent cardiac events, which could trigger a more intensive antithrombotic treatment by the treating physicians. However, this apparent paradoxical finding may be partially explained by a higher incidence of NSTEMI in the pretreatment group, a higher risk condition usually prompting an earlier and more aggressive antithrombotic treatment, although clinical severity at presentation (Killip class) did not differ.

In our study, clopidogrel was the most commonly used P2Y12i, followed by ticagrelor, whereas prasugrel was administered in only a small proportion of cases. The observed distribution likely reflects the extended inclusion period, which encompassed years when ticagrelor was not widely available or routinely used as first-line therapy. Furthermore, variability in drug availability across centers and in prehospital settings—particularly the limited accessibility of prasugrel—may also have influenced prescribing patterns. A statistically significant difference in aspirin use was also observed, with a substantially lower prescription rate among patients not receiving pretreatment. This finding may, in part, be explained by a lower prevalence of obstructive CAD in that group. Potential biases linked to center-specific practices and missing data must also be considered.

Similarly, differences in fondaparinux use may be attributed to its limited availability in some institutions, as well as possible selection bias: centers with access to fondaparinux, which is associated with lower bleeding risk [23], may have been less inclined to adopt a pretreatment strategy. Regarding the use of glycoprotein IIb/IIIa inhibitors, current guidelines suggest their use in the setting of high-risk PCI, particularly in patients who have not been pretreated with P2Y12i [9]. This may partly explain the observed difference in the use of these agents between groups.

Patients who received pretreatment exhibited higher rates of major bleeding, although the overall incidence of hemorrhagic complications in this cohort remained low and was lower than that reported in previous studies [21]. Patients in the pretreatment group more frequently received aspirin, ticagrelor (instead of clopidogrel), and unfractionated heparin/enoxaparin and more often underwent repeat CAG, all of which may have contributed to the observed increase in bleeding risk. It is also worth noting that this higher incidence of major bleeding may have been partially mitigated by the greater use of radial artery access in this group, a route associated with a lower risk of vascular and bleeding complications [24]. Notably, a recent observational study found that in-hospital bleeding not only influences discharge medication but also impacts long-term outcomes, including 1-year all-cause mortality, major bleeding, and re-infarction [25].

Taken together, these findings suggest limited evidence of benefit from routine pretreatment in NSTE-ACS patients undergoing a late invasive strategy, although causality cannot be established given the observational design of our study. It is important to note, though, that this analysis is limited to short-term, in-hospital events, and its relevance to long-term outcomes remains uncertain. Moreover, in specific NSTE-ACS subgroups—such as patients in resource-limited settings or those with delayed access to CAG, where guideline-recommended timings cannot be met and late invasive stratification frequently occurs >2 days after admission—pretreatment may still be a reasonable option. Further robust evidence from RCT conducted in contemporary real-world settings is needed to further clarify this issue and strengthen clinical recommendations, particularly regarding the late invasive strategy scenario.

### Limitations

The main limitation of this study relates to the extended inclusion period (2010–2023), which spanned multiple updates in clinical guidelines and consensus documents regarding P2Y12i pretreatment, potentially introducing heterogeneity in clinical management. While this extended timeframe was necessary to ensure an adequate sample size, it may have introduced temporal bias due to evolving practice patterns and drug availability. This heterogeneity over time should be considered when interpreting the results. Another important limitation is the use of in-hospital outcomes as endpoints, a decision driven by the available data in the registry, which may not accurately reflect clinical events occurring at 30 days or during long-term follow-up. In addition, several inherent limitations stem from the observational and voluntary nature of the registry, including the potential for missing or incomplete data, as well as the inability to exclude selection bias or residual confounding. The retrospective, non-randomized design also limits causal inference, particularly given possible differences in baseline therapy and disease severity between groups. Of note, data on fatal major bleeding events were unavailable, as such outcomes were not systematically captured in the registry, and the same applies to the type and subtype of major bleeding events, which were also not documented. Additionally, information on antiplatelet dosing, as well as on procedural complexity, was lacking, which may have limited the granularity of our analysis. A subgroup analysis according to the type of P2Y12i was not performed, as ticagrelor use in this cohort was relatively low and the overall number of bleeding events limited, which would have rendered such an analysis underpowered and unreliable. Information on acute stent thrombosis was also unavailable—an aspect worth considering in the context of pretreatment, given the findings of the ATLANTIC trial [5], which reported a slightly lower incidence in the pretreatment group, although the absolute difference between groups was minimal. However, considering the very low incidence of re-infarction in this cohort, substantial differences in stent thrombosis between groups are unlikely. Finally, although these findings are based on data from a Portuguese national registry, and thus may be influenced by local clinical practice, they are likely to have some degree of international relevance, given that Portuguese hospitals broadly adhere to European Society of Cardiology (ESC) guidelines. Nevertheless, regional variations in treatment within Portugal should still be considered when interpreting the results.

## 5. Conclusions

Baseline characteristics and clinical presentation of NSTE-ACS patients undergoing a late invasive strategy in this nationwide Portuguese registry were similar overall, irrespective of pretreatment status.

Our study showed that, in this population, pretreatment with a P2Y12i was not associated with a significant reduction in in-hospital endpoints but was linked to a higher incidence of major bleeding, which nonetheless remained low.

Although ESC guidelines clearly provide a weak recommendation for P2Y12i pretreatment in NSTE-ACS patients, real-world clinical practice is different. Registry-based studies such as ours contribute to a better understanding of this reality.

This article is a revised and expanded version of the abstract entitled “Prognostic implications of p2y12 inhibitor pretreatment in non-ST-segment elevation acute coronary syndromes undergoing late invasive strategy—a national registry analysis” [26], which was presented at ESC Congress 2024, London, from 30 August to 2 September 2024.

## Figures and Tables

**Table 1:** Patient baseline characteristics.

	Total (*n* = 3776)	Group 1 (*n* = 1530)	Group 2 (*n* = 2246)	*p*-Value
Male sex (%)	2691 (71.3)	1100 (71.9)	1591 (70.9)	0.494
Age, in years (mean ± SD)	66 ± 12	65 ± 12	66 ± 12	0.065
BMI, kg/m^2^ (mean ± SD)	27.8 ± 4.4	28 ± 4.4	27.8 ± 4.4	0.213
Hypertension (%)	2774 (74.0)	1108 (72.8)	1666 (74.8)	0.168
Dyslipidemia (%)	2326 (63.2)	894 (59.7)	1432 (65.7)	**<0.001**
Type 2 diabetes mellitus (%)	1286 (34.3)	514 (33.9)	772 (34.6)	0.638
History of smoking (%)	952 (25.2)	411 (26.9)	541 (24.1)	0.056
Heart failure history (%)	172 (4.6)	77 (5.0)	95 (4.3)	0.290
Valvular heart disease (%)	102 (2.7)	32 (2.1)	70 (3.2)	0.052
Prior angina (%)	1146 (30.8)	301 (19.8)	845 (38.4)	**<0.001**
Prior MI (%)	686 (18.8)	248 (16.3)	438 (20.5)	**0.001**
Prior PCI (%)	514 (13.8)	177 (11.6)	337 (15.4)	**0.001**
Prior CABG (%)	217 (5.8)	82 (5.4)	135 (6.1)	0.362
Prior ischemic stroke/TIA (%)	217 (5.8)	102 (6.7)	115 (5.2)	**0.003**
Peripheral artery disease (%)	197 (5.3)	76 (5.0)	121 (5.5)	0.533
Chronic kidney disease (%)	193 (5.5)	79 (5.2)	114 (5.7)	0.523
COPD (%)	186 (5.0)	93 (6.1)	93 (4.2)	**0.009**
Cancer history (%)	153 (4.7)	70 (4.6)	83 (4.8)	0.747
Dementia (%)	19 (0.6)	8 (0.5)	11 (0.7)	0.637
Prior history of bleeding (%)	48 (1.3)	16 (1.1)	32 (1.5)	0.264
Family history of CAD (%)	220 (6.2)	69 (4.9)	151 (7.0)	**0.011**

BMI—body mass index; CABG—coronary artery bypass graft; CAD—coronary artery disease; COPD—chronic obstructive pulmonary disease; MI—myocardial infarction; PCI—percutaneous coronary intervention; SD—standard deviation; TIA—transient ischemic attack. Statistically significant differences (*p* < 0.05) are presented in bold.

**Table 2:** Clinical, laboratorial, and echocardiographic parameters.

	Total (n = 3776)	Group 1 (n = 1530)	Group 2 (n = 2246)	*p*-Value
Admission diagnosis				
NSTEMI (%)	3295 (87.3)	1373 (89.7)	1922 (85.6)	**<0.001**
UA (%)	481 (12.7)	157 (10.3)	324 (14.4)	**<0.001**
Main symptom ^1^				
Acute chest pain (%)	3464 (92.5)	1394 (91.3)	2070 (93.3)	**0.024**
Dyspnea (%)	154 (4.1)	72 (4.7)	82 (3.7)	0.122
Cardiac arrest (%)	7 (0.2)	2 (0.1)	5 (0.2)	0.708
KK class ^1^				
KK class I (%)	3367 (90.1)	1364 (89.7)	2003 (90.3)	0.501
KK class > I (%)	371 (9.9)	157 (10.3)	214 (9.7)	0.501
Laboratory parameters ^1^				
Creatinine, mg/dL (mean ± SD)	1.1 ± 1	1.1 ± 0.9	1.1 ± 1	0.800
Hemoglobin, g/dL (mean ± SD)	13.9 ± 1.8	14 ± 1.8	13.8 ± 1.8	**0.003**
Platelets, ×10^3^/mm^3^ (mean ± SD)	220 ± 66	220 ± 66	220 ± 65	0.497
Echocardiographic parameters ^2^				
LVEF, % (mean ± SD)	56 ± 11	55 ± 11	57 ± 12	**0.001**
LVEF ≥ 50% (%)	2730 (77.7)	1129 (79.6)	1601 (76.5)	**0.032**
LVEF < 50% (%)	782 (22.3)	290 (20.4)	492 (23.5)	**0.032**

^1^ Evaluated at hospital admission; ^2^ evaluated during hospitalization. KK—Killip–Kimball; LVEF—left ventricular ejection fraction; NSTEMI—non-ST-segment elevation myocardial infarction; PCI—percutaneous coronary intervention; SD—standard deviation; UA—unstable angina. Statistically significant differences (*p* < 0.05) are presented in bold.

**Table 3:** Coronary angiography characteristics.

	Total (n = 3776)	Group 1 (n = 1530)	Group 2 (n = 2246)	*p*-Value
Time Admission-to-CAG in days—median [p25–p75]	2 [1,2,3]	2 [1,2,3,4]	2 [1,2,3]	0.852
Vascular access				
Radial artery (%)	2754 (78.6)	1231 (86.3)	1523 (73.3)	**<0.001**
Femoral artery (%)	751 (21.4)	196 (13.7)	555 (26.7)	**<0.001**
Normal CAG (no CAD) (%)	555 (14.7)	185 (12.1)	370 (16.5)	**<0.001**
Obstructive CAD (%)	2545 (79.9)	1162 (83.7)	1383 (76.9)	**<0.001**
Multivessel disease (%)	1726 (51.8)	746 (52.3)	980 (51.5)	0.667
Culprit vessel *				
LMA (%)	15 (1.3)	5 (1.1)	10 (1.4)	0.405
LAD (%)	324 (28.6)	118 (26.8)	206 (29.8)	**0.013**
LCx (%)	335 (29.6)	121 (27.4)	214 (30.9)	**0.005**
RCA (%)	459 (40.5)	197 (44.7)	262 (37.9)	0.851
More than one CAG during hospitalization (%)	196 (5.4)	121 (8.2)	75 (3.5)	**<0.001**
PCI (%)	2303 (61.0)	967 (63.3)	1336 (59.5)	**0.019**
Referral for CABG (%)	417 (11.1)	198 (13.0)	219 (9.8)	**0.002**

* When a culprit vessel was identified. CABG—coronary artery bypass graft; CAD—coronary artery disease; CAG—coronary angiography; LAD—left anterior descending artery; LCx—left circumflex artery; LMA—left main artery; PCI—percutaneous coronary intervention; RCA—right coronary artery. Statistically significant differences (*p* < 0.05) are presented in bold.

**Table 4:** In-hospital antithrombotic therapy.

	Total (n = 3776)	Group 1 (n = 1530)	Group 2 (n = 2246)	*p*-Value
Aspirin (%)	3247 (88.5)	1516 (99.1)	1731 (80.9)	**<0.001**
P2Y12 inhibitor				
Clopidogrel (%)	2234 (61.0)	1045 (68.4)	1189 (55.7)	**<0.001**
Ticagrelor (%)	1162 (31.9)	572 (37.8)	590 (27.7)	**<0.001**
Prasugrel (%)	8 (0.2)	2 (0.1)	6 (0.3)	1.000
Unfractionated heparin (%)	482 (13.2)	321 (21.1)	161 (7.6)	**<0.001**
Enoxaparin (%)	2405 (66.0)	1211 (79.7)	1194 (56.1)	**<0.001**
Fondaparinux (%)	851 (26.8)	183 (12.0)	668 (40.5)	**<0.001**
Glycoprotein IIb/IIIa inhibitors (%)	230 (6.3)	37 (2.4)	193 (9.1)	**<0.001**

Statistically significant differences (*p* < 0.05) are presented in bold.

**Table 5:** Primary and secondary in-hospital endpoints.

	Total (n = 3776)	Group 1 (n = 1530)	Group 2 (n = 2246)	*p*-Value	OR [CI 95%]
Primary composite endpoint (%)	330 (9.0)	134 (8.9)	196 (9.0)	0.906	0.99 [0.78–1.24]
Secondary endpoints					
All-cause mortality (%)	25 (0.7)	9 (0.6)	16 (0.7)	0.647	0.83 [0.36–1.87]
Non-fatal re-infarction (%)	36 (1.0)	20 (1.3)	16 (0.7)	0.072	1.82 [0.94–3.52]
Non-fatal stroke (%)	19 (0.5)	11 (0.7)	8 (0.4)	0.130	2.00 [0.80–4.97]
Heart failure (%)	280 (7.6)	107 (7.1)	173 (7.9)	0.353	0.89 [0.69–1.14]
Major bleeding (%)	17 (0.5)	12 (0.8)	5 (0.2)	**0.013**	**3.48** **[1.22–9.89]**

OR—odds ratio; CI—confidence interval. Statistically significant differences (*p* < 0.05) are presented in bold.

## Data Availability

The original contributions presented in this study are included in the article. Further inquiries can be directed to the corresponding author.

## Abbreviations

The following abbreviations are used in this manuscript:

ACS	Acute coronary syndrome
AF	Atrial fibrillation
CAD	Coronary artery disease
CABG	Coronary artery bypass graft
CAG	Coronary angiography
ECG	Electrocardiogram
ESC	European Society of Cardiology
HF	Heart failure
KK	Killip–Kimball
MI	Myocardial infarction
NSTE-ACS	Non-ST-segment elevation acute coronary syndrome
NSTEMI	Non-ST-segment elevation myocardial infarction
PCI	Percutaneous coronary intervention
ProACS	Portuguese registry of Acute Coronary Syndromes
P2Y12i	P2Y12 inhibitor
RCT	Randomized controlled trial
STEMI	ST-segment elevation myocardial infarction
TIA	Transient ischemic attack
UA	Unstable angina
